# Cytokine-Defined B Cell Responses as Therapeutic Targets in Multiple Sclerosis

**DOI:** 10.3389/fimmu.2015.00626

**Published:** 2016-01-08

**Authors:** Rui Li, Ayman Rezk, Luke M. Healy, Gillian Muirhead, Alexandre Prat, Jennifer L. Gommerman, Amit Bar-Or, MSSRF Canadian B cells in MS Team

**Affiliations:** ^1^Neuroimmunology Unit, Montreal Neurological Institute and Hospital, McGill University, Montreal, QC, Canada; ^2^Neuroimmunology Unit, Department of Neuroscience, Centre de Recherche du CHUM (CRCHUM), Université de Montreal, Montreal, QC, Canada; ^3^Department of Immunology, University of Toronto, Toronto, ON, Canada; ^4^Experimental Therapeutics Program, Montreal Neurological Institute and Hospital, McGill University, Montreal, QC, Canada

**Keywords:** multiple sclerosis, B-lymphocytes, cytokine-defined responses, immune modulation, B-cell depletion, B cell modulation

## Abstract

Important antibody-independent pathogenic roles of B cells are emerging in autoimmune diseases, including multiple sclerosis (MS). The contrasting results of different treatments targeting B cells in patients (in spite of predictions of therapeutic benefits from animal models) call for a better understanding of the multiple roles that distinct human B cell responses likely play in MS. In recent years, both murine and human B cells have been identified with distinct functional properties related to their expression of particular cytokines. These have included regulatory (Breg) B cells (secreting interleukin (IL)-10 or IL-35) and pro-inflammatory B cells (secreting tumor necrosis factor α, LTα, IL-6, and granulocyte macrophage colony-stimulating factor). Better understanding of human cytokine-defined B cell responses is necessary in both health and diseases, such as MS. Investigation of their surface phenotype, distinct functions, and the mechanisms of regulation (both cell intrinsic and cell extrinsic) may help develop effective treatments that are more selective and safe. In this review, we focus on mechanisms by which cytokine-defined B cells contribute to the peripheral immune cascades that are thought to underlie MS relapses, and the impact of B cell-directed therapies on these mechanisms.

## Introduction

**Figure 1:**
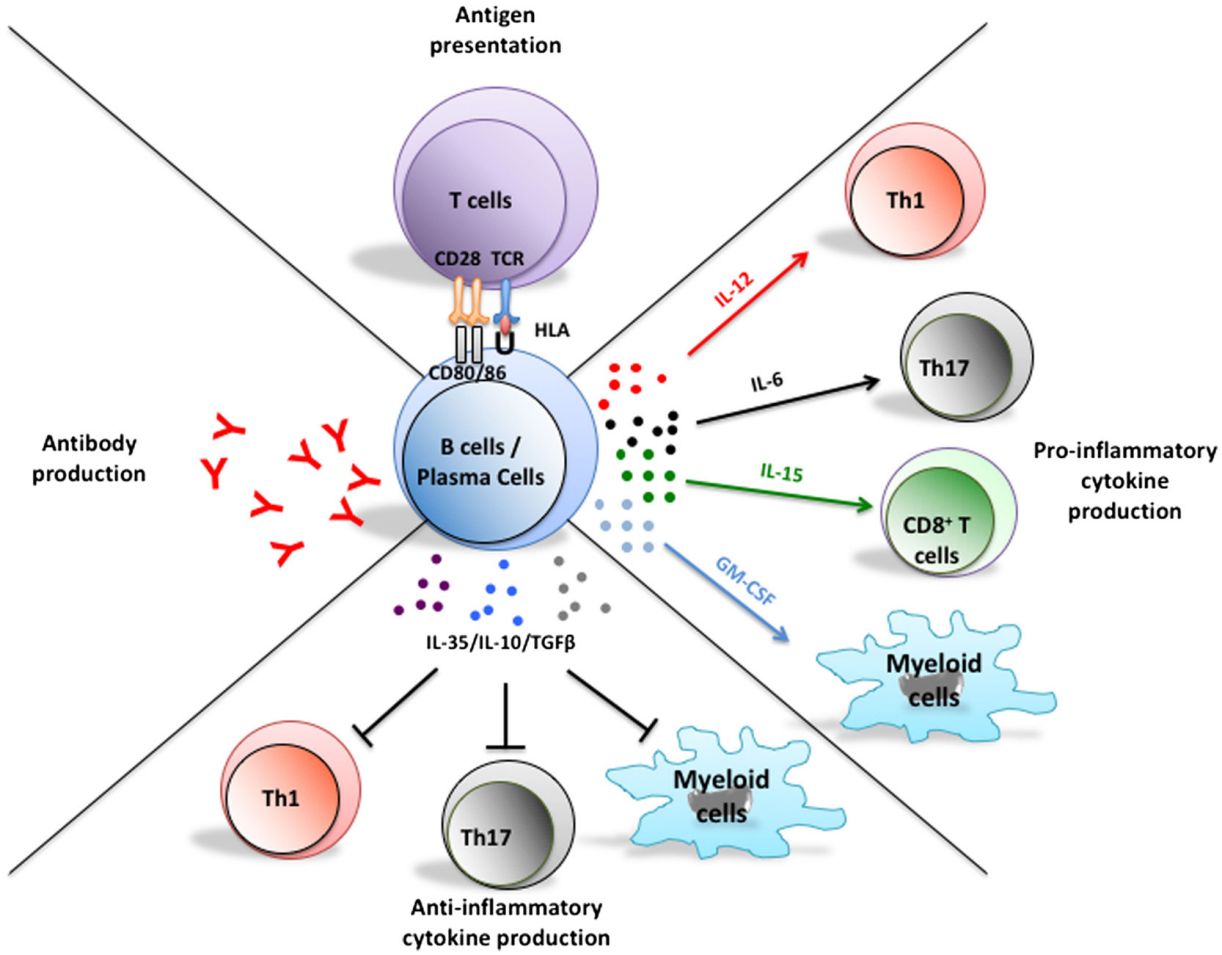
**Multifaceted functions of B cells and their implications in the pathogenesis of MS**. In addition to their potential to differentiate into antibody-secreting plasmablasts and plasma cells, B cells can present antigen to T cells, as well as up- or down-regulate local immune responses through elaboration of pro- or anti-inflammatory cytokine, respectively. Plasma cells can also secrete pro- and anti-inflammatory cytokines that could modulate T cell and myeloid-cell responses. Abnormal B cell responses of potential relevance to MS include aberrant production of autoreactive antibodies, exaggerated activation of T cells through antigen presentation, and induction of pro-inflammatory T cell and myeloid-cell responses through abnormal secretion of pro-inflammatory B cell cytokines and/or insufficient secretion of B cell regulatory cytokines.

In addition to their potential to differentiate into antibody producing plasma cells, B cells can efficiently present antigens to T cells and modulate local immune responses through secretion of soluble products, such as pro-inflammatory or anti-inflammatory cytokines (Figure 1) (1). Historically, B-cell implication in multiple sclerosis (MS) pathogenesis was based on the common finding of abnormally increased immunoglobulin levels in the cerebrospinal fluid (CSF) of patients as well as antibody deposition noted in brain lesions (2–5). However, the success of B-cell-depleting therapy to limit new MS relapses without obviously impacting abnormal CSF antibody levels underscores antibody-independent contributions of B cells to relapsing disease activity (6–11). In this review, we focus on implication of pro-inflammatory or anti-inflammatory cytokine-defined B cell responses in MS and the impact of B-cell-directed therapies on their functions.

## Cytokine-Defined B Cell Responses in MS and EAE

Several functionally distinct cytokine-defined B cell responses have been implicated in the pathophysiology of MS and its commonly used animal model, experimental autoimmune encephalomyelitis (EAE). Nonetheless, translation of B-cell-related findings from mouse to human has not always been straightforward. Characterizing and defining the roles of distinct human B cell subsets in health and disease are important requisites for rational development of more selective and effective B cell-targeting therapies.

## Interleukin-10-Producing B Cells

Interleukin (IL)-10 is a cytokine with pleiotropic effects in immunoregulation and inflammation (12). In mice, knock-out (KO) of IL-10 selectively from B cells results in more severe EAE (13), and adoptive transfer of *in vitro*-induced IL-10-producing B cells suppresses EAE in an IL-10-dependent manner (14–16). Inducing EAE in IL-10 reporter mice implicated the draining lymph nodes (rather than spleen or spinal cord) as the sites where IL-10^+^ B cells regulate disease-relevant immune responses (17). The IL-10^+^ B cells in this study exhibited plasma cell/plasmablast markers, consistent with an earlier report showing that CD138^+^ plasma cells are able to produce IL-10 (17), thus also highlighting the previously unappreciated antibody-independent functions of plasma cells. Although IL-10 production from B cells can be induced by both innate [toll-like receptors (TLRs)] and adaptive (cognate interaction) stimuli (14, 16, 18), the targets of regulation of innate- and adaptive-induced IL-10-producing B cells may differ depending on context. For example, innate signal-induced IL-10-producing B cells are able to down-regulate pathogenic T-cell responses indirectly through dendritic cells (14), whereas adaptive signal-induced IL-10-producing B cells directly down-regulate antigen-specific T-cell responses (15, 16).

In humans, both naïve and memory B cells are capable of producing IL-10 in a context-dependent manner (19–22). Human CD27^−^ (naïve) B cells, but not CD27^+^ (memory) B cells, are able to produce IL-10 upon CD40-ligand stimulation (11, 23–25), a response found to be abnormally deficient in B cells of MS patients (24). By contrast, IL-10^+^ B10 cells are induced within the CD27^+^ memory pool by stimulation through TLR4 and TLR9 and can suppress tumor necrosis factor α (TNFα) production by monocytes through an IL-10-dependent mechanism. Unexpectedly, B10 cells were reportedly increased in several human autoimmune diseases, including MS, upon stimulation (21). A better understanding of these cells, including defining surface markers and master transcriptional regulators, could facilitate future cell-based therapies for MS.

## IL-35 Producing B Cells

Interleukin-35 is an anti-inflammatory cytokine of the IL-12 family (26). Although the EBI3 subunit of IL-35 was first identified in EBV-infected B cells (27), functions of IL-35 were initially described in regulatory T cells (28–30). More recently, IL-35-producing B cells were found to play important roles in recovery from EAE and experimental autoimmune uveitis (31, 32). In these contexts, IL-35-producing B cells inhibited pro-inflammatory immune responses either directly through IL-35 (31) or indirectly through induction of IL-10-producing B cells (32). These IL-35-producing B cells also exhibited plasma cell phenotypic markers (31). Besides IL-10 and IL-35, B cells can also produce Transforming-growth factor β or Granzyme B that may down-regulate immune responses (33–39); their relevance to MS (or EAE) is yet to be determined.

## Tumor Necrosis Factor α and Lymphotoxin-α Producing B Cells

Tumor necrosis factor α and Lymphotoxin-α (LTα) are actively involved in promoting pro-inflammatory immune responses to protect against pathogen invasion (40). In addition, TNFα is also known to play a pathogenic role in several autoimmune diseases, including rheumatoid arthritis (41) and inflammatory bowel disease (42), in which TNFα-blocking therapies have been successful (41). In MS, however, TNFα blockade increased disease activity (43) highlighting the challenge of broadly targeting individual cytokines (versus targeting particular cytokine-expressing cells). Stimulation through CD40 and the B-cell receptor (BCR) significantly increases TNFα and LTα secretion from human B cells, compared to either stimulation alone (19). B cells of MS patients produce abnormally higher levels of both TNFα and LTα upon such dual stimulation (11, 23, 24). A microRNA (miR)-132:SIRT1 axis controls expression of TNFα and LTα by human B cells (23). Abnormally increased expression of miR-132 by MS B cells inhibited their SIRT1 expression, resulting in enhanced pro-inflammatory cytokine production. *In vitro* addition of the SIRT1-agonist resveratrol normalized the exaggerated pro-inflammatory cytokine expression of MS B cells (23).

## IL-6 Producing B Cells

Interleukin-6, a cytokine with both pro-inflammatory and anti-inflammatory properties, can be produced by both immune and non-immune cells (44). IL-6 can induce Th17-cell differentiation from naïve T cells (45) and inhibit regulatory T cells (46–48). By contrast, IL-6 may induce IL-10-producing regulatory B cells and myeloid cells (18, 49). B cells of MS patients secrete abnormally high levels of IL-6 (50) and IL-6 knock-out selectively from B cells resulted in decreased Th17 responses and diminished EAE severity (50, 51). How B cell-derived IL-6 is regulated, and whether B-cell IL-6 also contributes to Th17 differentiation and regulatory T-cell dysfunction in MS, remains unknown.

## IL-15 Producing B Cells

Interleukin-15 belongs to the four α-helix bundle family of cytokines and can be produced by multiple cell types (52). IL-15 knock-out mice develop more severe EAE (53), in part attributed to IL-15’s ability to inhibit pathogenic Th17-cell differentiation (54), and to induce regulatory CD8^+^ CD122^+^ T cells (55). In patients with MS, however, IL-15 is abnormally increased in both serum and CSF (56, 57), where it may have disease-promoting (rather than disease-inhibiting) potential (58, 59). B cells from MS patients reportedly produce more IL-15 than controls, and activation of B cells through CD40 and the BCR induces IL-15 secretion that enhanced both the migratory capacity of CD8^+^ T cells across a model of the blood–brain barrier and CD8^+^ T cell cytotoxicity toward oligodentrocytes (59).

## Granulocyte Macrophage Colony-Stimulating Factor-Producing B Cells

Granulocyte macrophage colony-stimulating factor (GM-CSF) is an important growth factor for myeloid lineage cell development and function, which is secreted by both immune and non-immune cells during infection and autoimmune disease (60). GM-CSF KO is resistant to active EAE induction (61), and GM-CSF KO Th17 cells fail to induce passive EAE (62–64). Since GM-CSF-producing T cells are reportedly increased in the circulation of MS patients (65–67), T cells have been thought to be the main source of GM-CSF of relevance to MS and EAE (65–68). A murine B-cell population generated from B1a cells, termed “innate response activator (IRA)” B cells (69), was described to produce GM-CSF and found to play a GM-CSF-mediated protective role during infections (69, 70), as well as a GM-CSF-mediated pathogenic role in atherosclerosis (71). In contrast to the murine IRA cells, a recently described human GM-CSF producing B cell subset belonged to the memory pool, and co-expressed high levels of TNFα and IL-6 (72). The human GM-CSF-producing B cells enhanced myeloid-cell pro-inflammatory responses in a GM-CSF-dependent manner and were abnormally increased in MS patients. B cell depletion in patients with MS resulted in a B cell–GM-CSF-dependent decrease of pro-inflammatory myeloid-cell responses, highlighting the potential pathogenic role of this B cell population *in vivo* and revealing a novel disease-implicated axis involving B cell:myeloid-cell interactions (72).

## B Cell-Targeting Therapies and Effects in MS

The use of B cell-depleting agents in MS was initially driven by the long-standing recognition of abnormal antibody presence in both the CSF and brain lesions of MS patients (2–4, 73). Therapies directed against B cells include agents that impact their survival (rituximab, ocrelizumab, ofatumumab, alemtuzumab, and atacicept), and their trafficking to the CNS (natalizumab and fingolimod). In this section, we will highlight the mechanisms of action of these and other MS-related therapies that may impact B cells, with a focus on how such therapies may influence MS disease-relevant cytokine-defined B cells responses.

## Anti-CD20 Monoclonal Antibodies

CD20 is a transmembrane protein with incompletely understood function, expressed on immature, transitional, naïve, and memory B cells, but not on stem cells, pro-B cells, and plasma cells (74). Rituximab, ocrelizumab, and ofatumumab are anti-CD20 monoclonal antibodies that induce B cell lysis via different combinations of antibody-dependent cell cytotoxicity, complement-dependent cytotoxicity, or apoptosis (75, 76). In MS, anti-CD20 antibodies rapidly and significantly reduced the number of new gadolinium-enhancing brain lesions and significantly reduced relapse rates (6–10, 77). Treatment reduced circulating B cell counts by >90% of baseline values, while serum and CSF immunoglobulin G levels remained largely unchanged (77–79), pointing to an important antibody-independent contribution of B cells to MS relapsing disease activity. An attractive hypothesis that has emerged is that pro-inflammatory B cells in untreated patients abnormally activate disease-relevant responses of other immune cells – hence removal of such B cells diminishes disease activity. In support of this view, anti-CD20-mediated B-cell depletion decreases both Th1 and Th17 T cell responses (11, 50) and pro-inflammatory myeloid-cell responses (that in turn could drive Th1 and Th17 responses) in the periphery of treated patients (72). In addition to cognate interactions in which B cells may serve as efficient antigen-presenting cells (APC) to activate T cells that recognize the same antigen, abnormal B-cell secretion of pro-inflammatory cytokines (including IL-6, TNFα, LTα, and GM-CSF) has now been implicated in abnormal T-cell and myeloid-cell responses of MS patients and may involve “bystander activation” (i.e., not be predicated by cognate antigen-specific interactions). Rituximab treatment could also diminish T cells within the CSF (79), providing further support that, when present, B cells may contribute to disease activity by enhancing peripheral T-cell activation and trafficking, and/or by CNS resident B cells promoting chemotaxis of T cells into the CNS. Alternative mechanisms proposed include an increased frequency of circulating regulatory T cells following B cell depletion (80) and in addition to depleting circulating B cells, anti-CD20 treatment also removes a small population of CD20^dim^ T cells (81, 82). Initial studies of this T cell subset point to their potential to produce pro-inflammatory cytokines (81), though their significance in relation to MS disease activity warrants further investigation.

Elegant work using somatic mutation analysis of the Ig gene in B cells derived from both CNS and peripheral compartments of the same MS patients indicates that bi-directional trafficking of B cells occurs between the CNS and periphery and that much of the activation and maturation that results in clonal enrichment of B cells within the CNS may actually occur in the periphery (presumably through cognate–antigen interactions with T cells) (83–86). Hence, efforts to deplete or modulate the profile and functions of B cells in peripheral compartments of MS patients may meaningfully influence the profile and activities of B cells within the CNS, even if the treatment itself does not efficiently penetrate the CNS (as is generally the case for monoclonal antibodies). There have also been efforts to selectively eliminate B cells in the CNS using intrathecally administered rituximab (87). However, a complication in interpreting this result has been the finding that even small doses of rituximab infused into the CSF results in rapid and substantial peripheral B cell depletion (87). Data regarding effects of anti-CD20-mediated peripheral B-cell depletion on inflammation within the CNS compartment remain limited. Early work suggested that rituximab may be more effective at depleting CSF B cells in patients with relapsing compared to progressive forms of MS (77, 78), possibly due to differences in Blood-brain barrier permeability. In the earlier OLYMPUS trial (88), rituximab failed to limit progression of disability in PPMS patients compared to placebo treatment, though *post hoc* sub-group analysis suggested that patients who had gadolinium-enhancing lesions at baseline, and particularly younger patients, did benefit (88). The ORATORIO study, focusing on earlier disease, and using ocrelizumab, demonstrated that anti-CD20 therapy could limit disease progression in PPMS patients (89). The mechanisms underlying this benefit of B cell depletion in patients with progressive MS remain to be elucidated (see Michel et al., in this issue).

In addition to the decreased MS disease activity observed following B cell depletion with anti-CD20 antibodies, there is a suggestion that the benefit of B cell depletion may persist in at least some patients even as reconstitution of B cells occurs (6, 7). This would imply that the re-emerging B cells differ importantly from the B cells present prior to depletion. Indeed, the B cells that reconstitute following anti-CD20 depletion have been shown to be largely naïve B cells which, when activated, express more IL-10 and less pro-inflammatory cytokines, including TNFα, IL-6, and GM-CSF, compared to pre-treatment B cells (72).

## Atacicept

B-cell activating factor of the TNF family (BAFF) and a proliferation-inducing ligand (APRIL) are expressed by a variety of immune and non-immune cells (90, 91). Both cytokines signal through transmembrane activator and cyclophilin ligand interactor (TACI) and B cell maturation antigen (BCMA), while only BAFF binds to BAFF-R (90, 91). Both play important roles in the survival, maturation, and function of B cells and plasma cells (92–94). BAFF can also promote differentiation and expansion of Th17 cells in models of infectious and autoimmune diseases (95). BAFF and APRIL levels are reportedly elevated in MS patients (96, 97), where they are highly expressed by peripheral blood monocytes and T cells. BAFF is also abnormally expressed by astrocytes within MS lesions (77–79, 98). Atacicept, a soluble, recombinant fusion protein containing the extracellular ligand-binding portion of TACI receptor and a modified Fc portion of human IgG, prevents binding of BAFF and APRIL to their receptors (99). Atacicept thereby limits survival of mature and activated B cells as well as antibody-secreting plasma cells but does not appear to target pro- or memory B cells (100, 101). Treatment with atacicept reduces circulating B cell counts (by 60–70%) and substantially reduces serum IgM and IgA (but to a lesser extent IgG) levels (100, 102–104). While emerging as beneficial in systemic lupus erythematosus, development of atacicept in MS was halted due to increased relapsing disease activity (100, 103–105). Why atacicept induced rather than limited new MS disease activity remains unknown but may reflect differential effects on functionally distinct B cell or plasma cell responses. Indeed, BAFF can induce IL-10-producing B cells and suppress the generation of IL-15^+^ B cells (106). Hence, the dysregulated cytokine balance in B cells of untreated MS patients may actually be aggravated by atacicept, leading to aberrant responses of disease-relevant immune cells, such as pathogenic T cells and myeloid cells.

## Approved MS Therapies That may Impact B Cell Responses

While most approved MS therapies were developed based on their presumed ability to target T cells, many of them are now understood to also impact B cells in potentially disease-relevant ways (Table 1). For example, interferon (IFN)-β decreases the frequency of CD80-expressing B cells in treated MS patients, which could in turn limit peripheral T cell activation (107). IFN-β also enhances the numbers of circulating transitional B cells (108) and, such as glatiramer acetate (GA), may result in an anti-inflammatory shift of B cell cytokine responses (109, 110). Both treatment with IFN-β and GA unexpectedly increased serum BAFF (111, 112), which would not only support certain aspects of B cells and plasma cells but may also shape the balance between cytokine-defined B cells, as described above. While natalizumab modifies frequencies of circulating B cells and plasma cells (Table 1) its effects, if any, on B cell cytokine-response profiles are largely unknown (113–115). It is noteworthy that *in vivo* treatment with natalizumab not only limits trafficking but may also modify T cell activation, which may reflect a role for VLA-4 during T cell interaction with APC, including B cells (113). Treatment with fingolimod (FTY720), an S1P receptor targeting agent, not only reduces the overall number of circulating B cells but can also modulate the cytokine-response profile of B cells in treated patients (116–120). Indeed, the proportion of peripheral blood memory B cells was reduced in fingolimod-treated patients, while the proportion of regulatory and transitional B cells was increased (117–120). The shift in circulating B-cell subsets was paralleled by changes in B-cell cytokine production, with reduced TNFα and enhanced IL-10 expression in B cells from fingolimod-treated patients (117, 120). Fingolimod treatment may also enhance migration capacity of regulatory B cells based on *in vitro* modeling of blood–brain barrier trafficking (120). Mitoxantrone, a chemotherapeutic agent that targets type II topoisomerase (121), preferentially reduces memory B cell counts and leads to a profile of circulating B cells with a less pro-inflammatory profile (24). Alemtuzumab, a humanized monoclonal antibody-targeting CD52, rapidly depletes multiple immune cell subsets (including B cells) followed by distinct kinetics of reconstitution (122). B cells reconstitute faster than T cells (within 3–6 months post-treatment), with naïve B cells dominating the re-emerging B cell pool. Such a treatment effect would be expected to induce a favorable shift in the balance between pro- and anti-inflammatory B cell responses (122, 123). Further studies are required to elucidate potential MS-relevant effects of dimethyl-fumarate (BG12) and teriflunomide on B-cell profiles, including cytokine responses (124, 125).

**Table 1:** **Selected therapies approved for (or under investigation for) multiple sclerosis, and their *in vivo* effects on the profiles and cytokine responses of B cells**.

Drug name	Main drug target(s)	Effects on peripheral B cell subsets	Changes in expression of B cell cytokines
IFN-β	IFN-βR	⇑ CD19^+^ B cells (108)	⇑ IL-10, TGF-β, IL-12p70, IL-27p28 (108, 109)
		⇑ CD19^+^CD24^++^CD38^++^ B cells (108)	⇓ IL-1β, IL-23p19/40 (108, 109)
		⇓ % CD19^+^CD38^−^IgM^−^IgD^−^ (108)	
		⇓ % CD80^+^ B cells (107, 109)	
		⇓ % CD40^+^ B cells (109)	
Glatiramer acetate	MHC class II (126)	⇓ CD19^+^ B cells (127)	⇑ IL-10, IL-6 (127)
		⇓ % CD27^−^ B cells (128)	⇓ LTα (127)
Natalizumab	Alpha-4-integrin	⇑ % CD19^+^ B cells (114–116, 129)	Unknown
		⇑ CD19^+^CD10^−^CD138^−^ B cells (130)	
		⇑ CD19^+^CD10^+^ pre-B cells (130)	
		⇑ % CD27^+^IgD^+^ B cells (114)	
		⇓% CD27^−^IgD^+^ B cells (114)	
		⇑ % CD27^+^IgD^−^ B cells (114)	
Mitoxantrone	Type II topoisomerase (121, 131)	⇓ CD19^+^ B cells (132)	⇑ IL-10 (24)
		⇓ % CD27^+^ B cells (24)	⇓ LTα (24)
Fingolimod	S1P1R	⇓ CD19^+^ B cells (116–119)	⇑ IL-10, ⇓TNFα (117, 120)
		⇓ % CD27^+^ CD38^int-low^ B cells (117, 120)	
		⇑ % CD27^−^ B cells (117, 119, 120)	
		⇑ % CD38^+^CD27^−^CD24^+^CD5^+^ B cells (120)	
		⇑ % CD10^+^CD38^hi^CD24^hi^ B cells (117)	
Dimethyl-fumarate	Nrf2 (133)	⇓ CD19^+^ B cells (124, 125)	Unknown
Teriflunomide	Mitonchondrial enzyme dihydroorotate dehydrogenase (DHODH) (134)	⇓ Proliferation of T cells and B cells	Unknown
		⇓ Antibody titers against neoantigen but not recall antigens (135, 136)	
Alemtuzumab	CD52	⇓ CD19^+^ B cells (123, 137, 138)	May result in shift in the balance between pro- and anti-inflammatory cytokine networks in B cells
		⇑ CD19^+^CD23^−^CD27^–^ (after 1 month) (137)	
		⇑ CD19^+^CD23^+^CD27^−^ (after 3–12 months) (137)	
		Partial reconstitution of CD19^+^CD23^+^CD27^+^ B cells (after 12 month) (137)	
		⇑ CD19^+^CD24^hi^CD38^hi^ (at 6 months) (123)	
Rituximab	CD20	⇓ CD19^+^ B cells (but not plasma cells)	⇓ IL-6, TNFα, LTα
Ocrelizumab	Ofatumumab	Early reconstitution of CD27^−^B cells and CD19^+^IgD^+^CD38^hi^CD10^lo^CD24^hi^ B cells (6–10, 79, 98)	⇓ GM-CSF
			⇑ IL-10 (11, 24, 50)
Daclizumab	IL-2R-α	⇓ CD19^+^ B cells (139)	Unknown
		No change in CD19^+^ B cells (140)	
Atacicept^a^	BAFF/APRIL	⇓ % mature B cells and plasma cells (not memory B cells) (101, 103, 105)	Unknown but may result in ⇓ IL-10 and IL-35

*^a^Clinical trial program of atacicept in MS was discontinued when early studies indicated treatment resulted in increased disease activity (103)*.

## Conclusion

The success of anti-CD20 therapy in MS establishes that B cells contribute to relapsing disease activity. Though unwelcome, the observation that treatment with atacicept (Table 1) exacerbates MS, serves to reinforce the concept that targeting B cells can change the face of CNS disease activity, while also underscoring the importance of elucidating the functional heterogeneity that exists within the B cell pool. Emerging studies indicate that responses of cytokine-secreting B cells in the periphery may influence new MS disease activity, potentially through aberrant peripheral activation of other immune cells. B cells may play additional roles in propagating disease activity within the CNS (see Michel et al., in this issue). Success of B cell targeting therapies may lie in restoring and maintaining a favorable balance between pro- and anti-inflammatory B cell activities in patients.

## Conflict of Interest Statement

Rui Li, Ayman Rezk, Luke M. Healy, Gillian Muirhead, Alexandre Prat, and Jennifer L. Gommerman declare that the research was conducted in the absence of any commercial or financial relationships that could be construed as a potential conflict of interest. Dr. Bar-Or has participated as a speaker in meetings sponsored by and received consulting fees and/or grant support from Biogen Idec, Diogenix, Genentech, Sanofi-Genzyme, GlaxoSmithKline, Novartis, Ono Pharma, Teva Neuroscience, Receptos Inc, Roche, and Merck/EMD Serono.

The Handling Editor, Jorge Ivan Alvarez, declares that, despite having been supervised by author Alexandre Prat during post-doctoral work, the review process was handled objectively.
